# An array of signal-specific MoYpd1 isoforms determines full virulence in the pathogenic fungus *Magnaporthe oryzae*

**DOI:** 10.1038/s42003-024-05941-z

**Published:** 2024-03-04

**Authors:** Sri Bühring, Antonia Brunner, Klemens Heeb, Marius-Peter Mergard, Greta Schmauck, Stefan Jacob

**Affiliations:** 1 Institute of Biotechnology and Drug Research gGmbH (IBWF), Hanns-Dieter-Hüsch-Weg 17, 55128 Mainz, Germany; 2 Johannes Gutenberg-University Mainz, Microbiology and Biotechnology at the Institute of Molecular Physiology, Hanns-Dieter-Hüsch-Weg 17, 55128 Mainz, Germany

**Keywords:** Pathogens, Molecular biology

## Abstract

*Magnaporthe oryzae* is placed first on a list of the world’s top ten plant pathogens with the highest scientific and economic importance. The locus MGG_07173 occurs only once in the genome of *M. oryzae* and encodes the phosphotransfer protein MoYpd1p, which plays an important role in the high osmolarity glycerol (HOG) signaling pathway for osmoregulation. Originating from this locus, at least three *MoYPD1* isoforms are produced in a signal-specific manner. The transcript levels of these *MoYPD1-*isoforms were individually affected by external stress. Salt (KCI) stress raised *MoYPD1_T0* abundance, whereas osmotic stress by sorbitol elevates *MoYPD1_T1* levels. In line with this, signal-specific nuclear translocation of green fluorescent protein-fused MoYpd1p isoforms in response to stress was observed. Mutant strains that produce only one of the MoYpd1p isoforms are less virulent, suggesting a combination thereof is required to invade the host successfully. In summary, we demonstrate signal-specific production of MoYpd1p isoforms that individually increase signal diversity and orchestrate virulence in *M. oryzae*.

## Abstract (web summary)

This study suggests that signal-specific production of protein isoforms by alternative splicing individually increases diversity of signaling networks and orchestrates virulence in pathogenic fungi.

## Introduction

*Magnaporthe oryzae* is a filamentous phytopathogenic fungus which is extremely destructive to the cultivated crop rice (*Oryza sativa*) and ranks number one among the most important plant pathogens worldwide^1^. The facultative pathogen is hemibiotrophic, changing its lifestyle from bio- to necrotrophy during host invasion^2^. Its importance is highlighted based on the fact that almost half of the world’s population needs rice as a major food source. Even though the rice blast disease is being combated with great intensity, the pathogen annually still destructs crops that could feed more than 60 million people^3^. In this respect, a better understanding of this pathogen is a prerequisite to face the global food supply. *M. oryzae* is a wonderful organism to study the molecular basis of pathogenicity, since genome and transcriptome sequences of multiple strains are available and its genome is quite suitable for directed genetic manipulation. Although the significance of alternative splicing (AS) in the rice blast fungus has already been highlighted in a few studies, details about the function of individual isoforms regarding virulence have not yet been described. All statements on virulence to date have been made based on mutants in which only the entire genomic sequence of the genes of interest have been deleted^4–6^. There are no studies in which mutants have been created that produce only single individual isoforms.

**Fig. 1:**
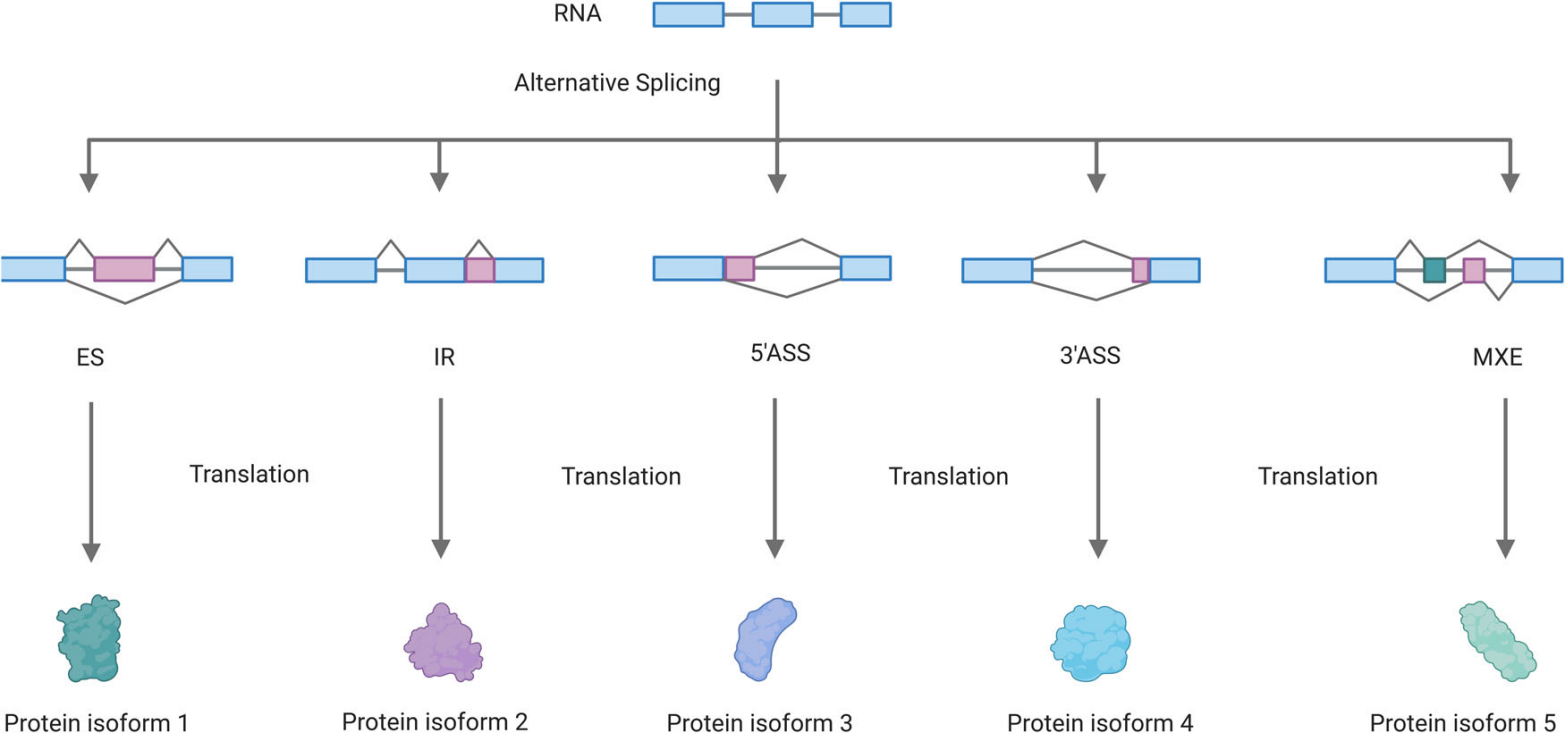
Simplified illustration of the five major alternative splicing (AS) patterns. The exons are represented as boxes and the introns as lines. The AS of the pre-mRNA via exon skipping (ES), intron retention (IR), alternative 5′ or 3′ splice sites (5′ ASS, 3′ ASS) and mutually exclusive exons (MXE) can produce different proteins.

Transcript identification and gene expression quantification have become core activities in molecular biology since the discovery of RNA’s key role as an intermediate between the genome and the proteome^7^. After the transcription of genomic DNA in eukaryotes, a protein complex called spliceosome removes introns from pre-mRNA and joins adjacent exons processing the mature mRNA^8–10^. Based on one genomic sequence, the spliceosome produces different mRNA transcripts through AS, which differ in stability, localization and coding sequences (CDS)^11,12^. Consequently, several protein-coding transcript variants with different or even opposite functions can be produced from a single gene. Therefore, AS is a fundamental mechanism in eukaryotes and enhances the regulatory and functional diversity of proteins and phenotypic traits^12,13^. The diversity of AS events are classified into five main categories (Fig. 1): the removal of a single exon (exon skipping), the retention of one intron in the mRNA (intron retention), alternative 5′ or 3′ splice sites and mutually exclusive exons.

Intron retention is the most commonly described AS event in fungi^14–16^. Even though the AS phenomenon has been known for over 40 years, the annotated transcripts only partially reflect the huge complexity of all AS events in fungi. Missing or incorrect genome annotations and the availability of datasets only about the most popular gene transcripts in the fungal databases limit our current knowledge of the biological impact of AS^17^. Increasing the analysis of RNA-seq data suggests a critical role for AS in the fungal kingdom^14–16^. The histidine-containing phosphotransfer protein Ypd1p plays an important role in osmoregulation as part of the high osmolarity glycerol (HOG) pathway. The latter is also involved in the regulation of growth, development and virulence in the rice blast fungus *M. oryzae*, and overstimulated by the phenylpyrrole fungicide fludioxonil^18–20^. The signaling pathway is composed of a multistep phosphorelay (MSP) system followed by a mitogen-activated protein kinase (MAPK) cascade downstream. The MSP system in *M. oryzae* comprises the hybrid sensor histidine kinases (HKs) MoHik1p and MoSln1p, the histidine-containing phosphotransfer (HPt) protein MoYpd1p and the response regulator (RR) MoSsk1p. The detailed molecular mechanisms of signal transfer is not well understood in filamentous fungi so far. In eukarytotic organisms, it is generally assumed that MSP signal phosphoryl group transfer is mediated by the His-Asp-His-Asp manner^21,22^. In fungi, there are only few evidence-based studies, mostly using the baker yeast *Saccharomyces cerevisiae* as model organism^23^. According to homology-based assumption for the components of the MSP in *M. oryzae*, we presume that in response to changing environmental osmolarity, MoYpd1p may act as the central linchpin and transmits signals through reversible phosphorylation and dephosphorylation within the MSP. Under isosmotic conditions, the MSP system may be constitutively phosphorylated, thereby, inhibiting the MAPK cascade. By contrast, the signaling components are assumed to be dephosphorylated as external osmolarity rises, which results in the activation of the MAPK cascade by phosphorylation^24^. Finally, the phosphorylated MoHog1p translocates into the nucleus and induces the stress response^25^. Compared to most fungal genes, only one *MoYPD1* gene is annotated in the genome, with two transcripts (*MoYPD1_T0* and *MoYPD1_T1*). It is well-known that MoYpd1p interacts with MoSln1p and MoHik1p in the MSP^26^. Apart from MoSln1p and MoHik1p, eight further HKs are encoded in the genome of *M. oryzae*, which may perform protein-protein interactions (PPI) with MoYpd1p^19^. Moreover, a third transcript isoform *(MoYPD1_T3)* was recently identified on the cDNA and protein level^27^. In addition to its key role in the HOG signaling pathway, *MoYPD1* appears to positively and negatively influence genes involved in other stress adaptation processes and virulence^6^. Studies on Ypd1p homologs in other fungi, such as *Candida albicans* and *Aspergillus fumigatus*, have shown that green fluorescent protein (GFP)-fused Ypd1p shuttles between the cytoplasm and the nucleus^28,29^. Therefore, it is conceivable that MoYpd1p interacts with targets in both the cytosol and nucleus. However, the specific function of *MoYPD1* isoforms and their potential for signal-specific functions still remains enigmatic. A better understanding of the complex role of *MoYPD1* in *M. oryzae* can be obtained by the identification and characterization of additional isoforms. We used various bioinformatics tools and validated the predictions in molecular biology experiments to answer the questions of which *MoYPD1* transcript isoforms exist and whether they are produced or function in a signal-specific manner in the HOG pathway.

## Results

### Signal-specific localization of MoYpd1p isoforms and their role in pathogenicity

In order to identify and follow spatial localization of the different phosphotransferase isoforms MoYpd1p_T0, MoYpd1p_T1 and MoYpd1p_T2 (see Supplementary Table S2), we generated different mutant strains by fusing a GFP to the genomic sequence of *MoYPD1* (referred to on a protein level as total MoYpd1p) and to isoform-specific cDNA sequences (MoYpd1p_T0, MoYpd1p_T1, MoYpd1p_T2, Supplementary Table S3). The loss-of-function mutant *ΔMoypd1* was used as the parent strain. Consequently, we have the mutant strain *ΔMoypd1::MoYPD1-GFP* producing “all” isoforms, and the mutant strains *ΔMoypd1::MoYPD1_T0-GFP, ΔMoypd1::MoYPD1_T1-GFP* and *ΔMoypd1::MoYPD1_T2-GFP* producing only the single isoforms MoYpd1p_T0, MoYpd1p_T1 and MoYpd1p_T2, respectively.

**Fig. 2:**
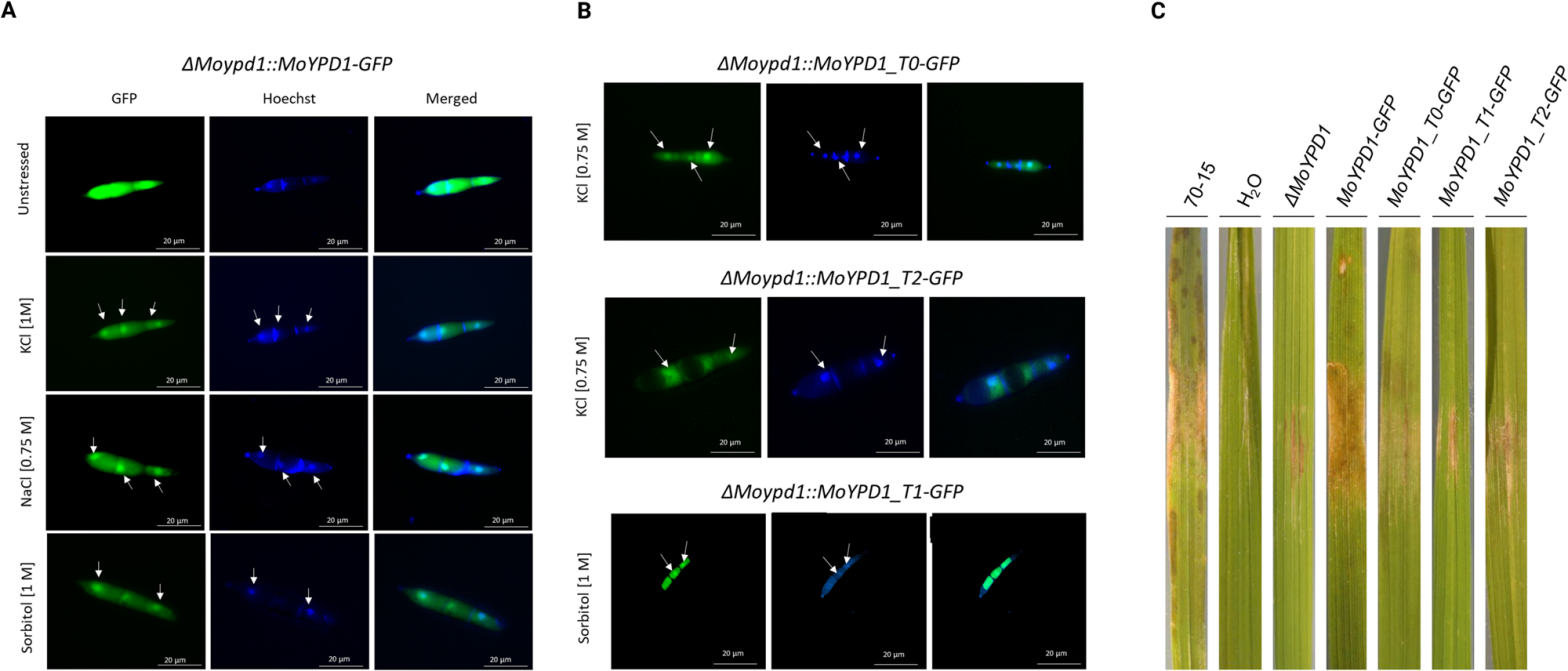
Localization of GFP-fused total MoYpd1p and different MoYpd1p isoforms. **A** Translocation of GFP-fused total MoYpd1p 1 min after salt (KCl [1 M] and NaCl [0.75 M]) or sorbitol [1 M] treatment. The GFP signal in the untreated control was distributed throughout the cytoplasm of the mutant strain ΔMoypd1::GFP-MoYPD1. **B** Localization of GFP-fused MoYpd1p isoforms. After treatment with KCl [0.75 M], the translocation into the nucleus was observed for MoYpd1p_T0 and MoYpd1p_T2. However, MoYpd1p_T1 was found to accumulate in the nucleus upon sorbitol stress [1 M]. **C** Lesions on rice leaf surfaces three days after inoculation with M. oryzae. Detached rice leaves were inoculated with a conidial suspension of the wildtype strain 70-15, ΔMoypd1::MoYPD1-GFP, ΔMoypd1::MoYPD1_T0-GFP, ΔMoypd1::MoYPD1_T1-GFP or ΔMoypd1::MoYPD1_T2-GFP. Mutant strains producing only one of the isoforms were found to cause fewer disease symptoms on the leaves compared to the control strains.

The GFP signal in all mutant strains was observed to be distributed within the cytoplasm under isoosmotic conditions, whereas the respective subcellular localization of total MoYpd1p and the three isoforms was found in the nucleus in response to different stress stimuli. In detail, we found an accumulation of the GFP-signal of total MoYpd1p in the nuclei of the mutant strain *ΔMoypd1::MoYPD1-GFP* one minute after stress exposure to KCl [1 M], NaCl [0.75 M] or sorbitol [1 M] (Fig. 2A, time-course in Supplementary Fig. S1). By contrast, the spatial localization of MoYpd1p_T0, MoYpd1p_T1 and MoYpd1p_T2 changes individually after stress, providing a deeper understanding of the role of AS in signal diversity within phosphorelay systems.

The respective isoforms MoYpd1p_T0 and MoYpd1p_T2 translocate into the nucleus in the mutant strains *ΔMoypd1::MoYPD1_T0-GFP* and *ΔMoypd1::MoYPD1_T2-GFP* as a result of 0.75 M KCl stress, whereas the isoform MoYpd1p_T1 in the mutant strain *ΔMoypd1::MoYPD1_T1-GFP* was located within the nucleus in response to sorbitol treatment (Fig. 2B). After exposure to sorbitol stress [1 M], the GFP-signal of both isoforms MoYpd1p_T0 and MoYpd1p_T2 remained in the cytoplasm (Supplementary Fig. S2).

The role of MoYpd1p and its isoforms in virulence is shown in Fig. 2C. The wildtype strain 70-15 was found to cause significant lesions on detached rice leaves, whereas the mutant strains producing only one of the isoforms MoYpd1p_T0, MoYpd1p_T1 or MoYpd1p_T2 have been documented to be less virulent. The positive control of the complemented mutant strain *ΔMoypd1::MoYPD1-GFP* shows similar infection phenotypes as compared to the wildtype strain, indicating that all isoforms collectively are needed for full virulence in *M. oryzae*. Experiments on whole rice plants inoculated with conidial suspensions underlined this hypothesis. Similar to the detached leaf assays, the same results could be observed in the plant assays (Supplementary Fig. S3A).

In line with this, a vegetative growth assay validated the need for an interplay of all MoYpd1 isoforms in order to achieve a completely functional osmoregulation in *M. oryzae* (Supplementary Fig. S3B). MoYpd1p-producing isoform strains showed growth inhibition of over 70% in the presence of high concentrations [0.4 and 0.6 M] of KCl or sorbitol. No significant difference in growth between the mutant strains producing only one of the isoforms MoYpd1p_T0, MoYpd1p_T1 or MoYpd1p_T2 has been documented. That strongly suggests that individual isoforms are not sufficient to cope with conditions of sustained high osmolarity.

### Additional *MoYPD1* isoforms and signal specific splicing

**Fig. 3:**
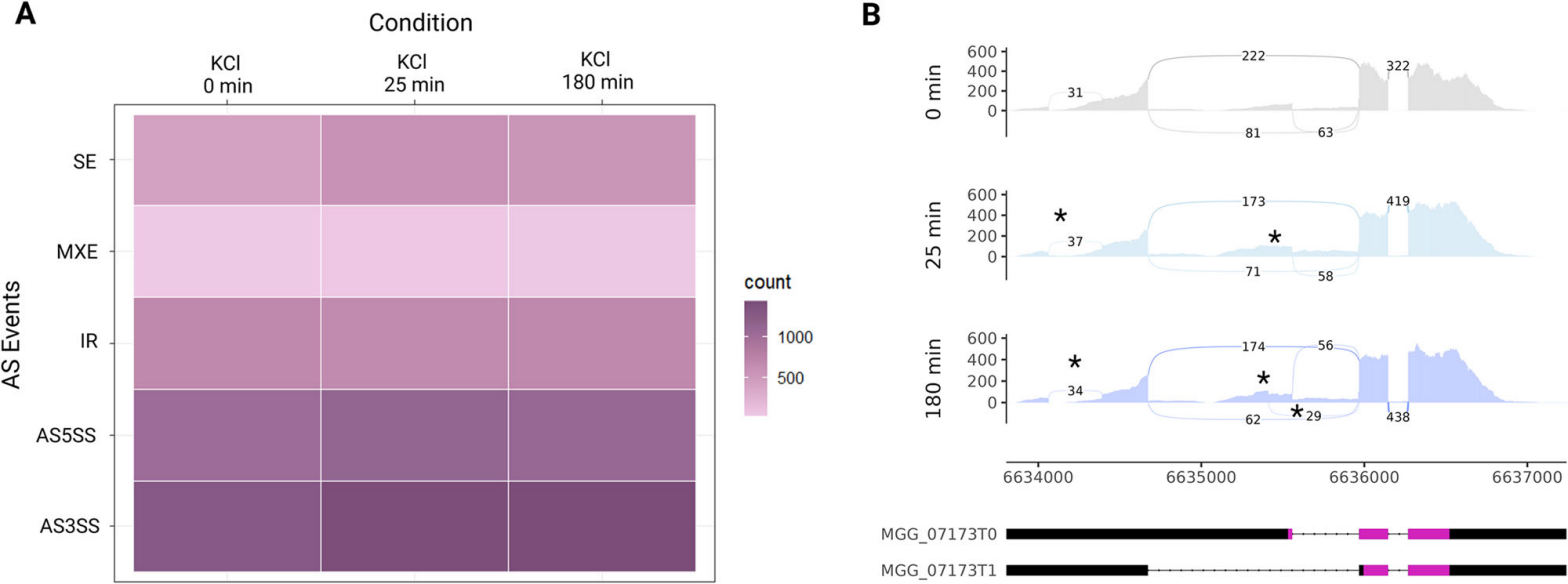
Evaluation of RNA-seq data. **A** Heat map of RNA-Seq transcriptome analysis for AS Events from M. oryzae under unstressed and stressed conditions (0 min, 25 min and 180 min after treatment with 0.5 M KCl. 3’ ASS and 5’ ASS were found to be the most common AS patterns following KCl stress induction. **B** Sashimi plot visualized the AS pattern of MoYPD1 during 0.5 M KCl stressed and unstressed conditions. In addition to the annotated introns of MGG_07173T0 and MGG_07173T1, additional splice junctions are shown (see asterisk).

As a result of our replicate multivariate analysis of transcript splicing (rMATS) analysis, we were able to determine the total number of AS events of protein-coding genes in *M. oryzae* between the unstressed condition and KCl [0.5 M], sorbitol [0.5 M] and fludioxonil-stressed [10 µg/ml] conditions. In this context, 3’ ASS (about 37%) and 5’ ASS (approximately 30%) were identified as the most common AS patterns in all samples. The total number of AS events after exposure to KCl increased from 3474 to 3851. The number of AS events over the same period after sorbitol exposure were found to be decreased from 3661 to 3292, and from 3474 to 2766 after treatment with fludioxonil (Fig. 3A, Supplementary Table S4). How the stress conditions tested influence *MoYPD1* splicing patterns is shown in Sashimi plots (Fig. 3B).

The coverage tracks in the Sashimi plots are illustrated in gray, light blue and purple, with exons connected by arcs indicating splicing events. Annotated transcripts of isoform *MoYPD1_T0* and isoform *MoYPD1_T1* are presented below the Sashimi plots, where pink represents the CDS. In addition to the stress-independent splicing events of the annotated isoforms and an additional splice junction (Chr2:6634064-6634391) in the 5’ direction of the CDS, unannotated splice sites were also revealed by KCl exposure (marked by an asterisk). A similar splicing pattern was observed upon sorbitol and fludioxonil-induced stress (Supplementary Figs. S4 and S5). Based on our rMATS analysis, four possible new *MoYPD1* isoforms with alternative splice regions have been identified. We detected the isoforms using event- and isoform-based prediction tools and underpinned this with an IsoSeq-based transcript assembly (Table 1).

**Table 1:** Selection of different *MoYPD1* transcript isoforms using different prediction tools

Tools						
rMATS	✓	✓	✓	✓	✓	✓
Cufflinks	✓	✓	–	–	✓	–
IsoSeq	✓	✓	–	–	✓	–
MAJIQ+Voila	✓	✓	–	✓	✓	✓
SGSeq	✓	✓	–	✓	✓	✓

The in silico analysis contributed 5’GT-AG 3’ as a splice site; this motif is known to be a critical factor for properly splicing RNA in eukaryotes. The region between the two posterior exons is a conserved region of the isoforms; differences in splice patterns result from the length of the first intron. An exception is the isoform (isoform T4) mentioned previously with an additional exon. The *MoYPD1* isoforms T0, T1 and T4 were consistently detected by all methods, indicating their widespread presence and biological importance. However, even though rMATS, modeling alternative junction inclusion quantification (MAJIQ)+Voila and SGSeq predicted the existence of the transcript *MoYPD1* isoforms T3 and T5, only rMATS detected the presence of *MoYPD1_T2*, an isoform already determined at the peptide level^27^. After the prediction of various isoforms for *MoYPD1*, the *MoYPD1* isoforms T1–T5 were amplified at the cDNA level and confirmed by sanger sequencing. The ORF prediction using NCBI ORF Finder reveals a novel ORF for isoform T3 in addition to the ORFs already known (Table 2).

**Table 2:**
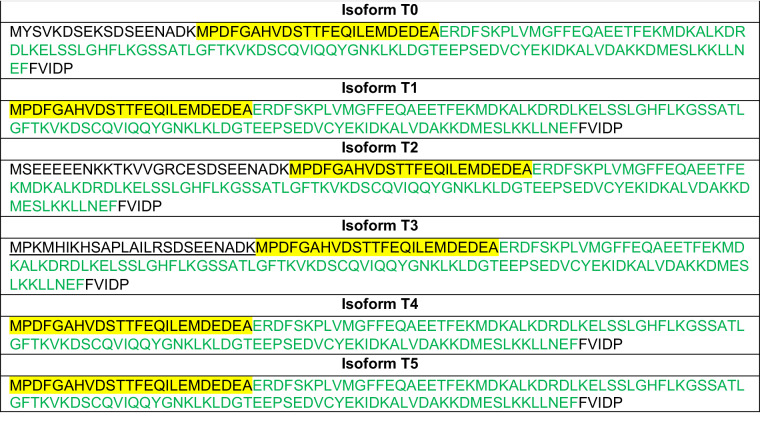
Predicted ORFs of *MoYPD1* transcript isoforms

The underlined markers highlight the unannotated sequences of the ORF of isoform T3. The Hpt domain from uniprot (G4MTK9) is given in green, whereas the additionally 24 AS found in all isoforms are given in yellow.

A core sequence beginning with MPDFGAHV and ending with FVIDP is present in all ORFs of the isoforms, whereas its predicted Hpt domain is 24 AS longer compared to the annotated Hpt domain from uniprot (G4MTK9). Interestingly, FVIDP is not part of the Hpt domain. The N-terminal sequences upstream of the core sequence of isoforms T0, T2 and T3 differ by 18aa (isoform T0), 27aa (isoform T2) and 25aa (isoform T3), respectively. The conserved sequence suggests it forms the typical four-helix bundle of the Hpt domains of the MoYpd1p isoforms. The prediction of different splicing patterns for the isoforms T1, T4 and T5 based on RNA-seq data despite identical ORFs of the three transcript isoforms suggests that differences in the non-coding regions of the 5’ or 3’ UTRs may contribute to functional differences.

**Fig. 4:**
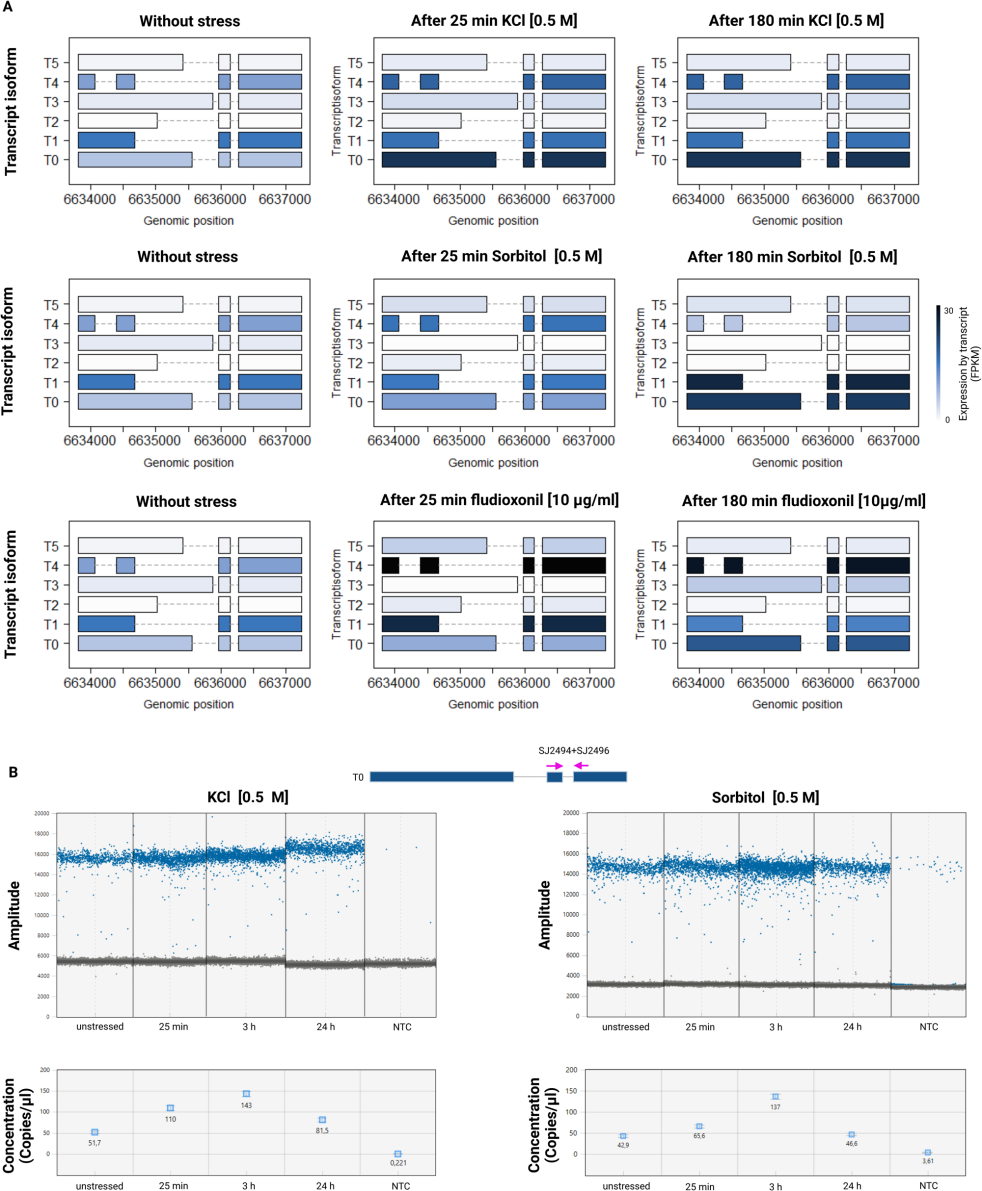
*MoYPD1* isoform expression analysis. **A** (Top): Heat plot of *MoYPD1* isoform expression under different conditions. Isoosmotic conditions result in the highest FPKM values for *MoYPD1_T1*. The expression level of isoform T0 after 25 and 180 min of 0.5 M KCl treatment exceeds that of *MoYPD1_T1*, resulting in *MoYPD1_T0* being the dominantly expressed isoform. However, the transcript level of isoform T1 is highest after sorbitol stress (middle). The fludioxonil triggered expression is also different since isoform T4 is produced at the highest level. **B** ddPCR result of the conserved *MoYPD1* region. Top:1 D plot of ddPCR reaction under KCl- and sorbitol-stressed conditions. A blue dot indicates a positive droplet containing at least one copy of *MoYPD1* and a gray dot indicates a negative droplet lacking any target DNA. Bottom: Concentrations (copies/μl) of the conserved *MoYPD1* region, as processed by QuantaSoftware™. The error bars show the maximum and minimum Poisson distributions for the 95% confidence interval.

Isoform expression analyses were performed with DESeq2 during the next step in order to investigate potential signal-specific functions of the isoforms. The most prevalently expressed isoform under non-stressed conditions was T1, followed by T0 and T4. According to our findings, the transcript *MoYPD1* isoforms T2, T3 and T5 exhibited a consistently low-frequency expression, whereas the FPKM (Fragments Per Kilobase of transcript per Million mapped reads) values of the remaining isoforms change depending on the stress induction. The expression of *MoYPD1_T0* increases after treatment with 0.5 M KCl, whereas the expression of *MoYPD1_T1* increases after treatment with 0.5 M sorbitol or 10 µg/ml fludioxonil (Fig. 4A). Additionally, it was found that fludioxonil triggers much more strongly the expression of isoform T4 as compared to KCl or sorbitol stress.

In conclusion, these observations demonstrate that the isoforms T0 and T1 are dominantly produced under isoosmotic conditions. Stress induces the translocation of these proteins into the nucleus and, simultaneously, increases their expression level. Furthermore, the results are underpinned using droplet digital polymerase chain reaction (ddPCR) analysis investigating the absolute expression pattern (Fig. 4B). In order to amplify all possible isoforms, we used the primer combination SJ2494 and SJ2496 (Supplementary Table S5), capable of amplifying the conserved region in the gene. As a result of KCl and sorbitol stress, we observed a nearly 2- and 3-fold increase in copies/µl, respectively, at 25 and 180 min for the conserved *MoYPD1* region, which decreased to pre-stress levels after 24 h. Taken together, these results confirm our hypothesis of the existence of more isoforms of *MoYPD1* than previously described. Additionally, we provide evidence for signal-specific changes of the expression levels of isoform transcripts and different signal-specific subcellular localizations of isoform proteins upon KCl, sorbitol and fungicide stress, respectively.

### A new model of the HOG pathway comprises more elements

**Fig. 5:**
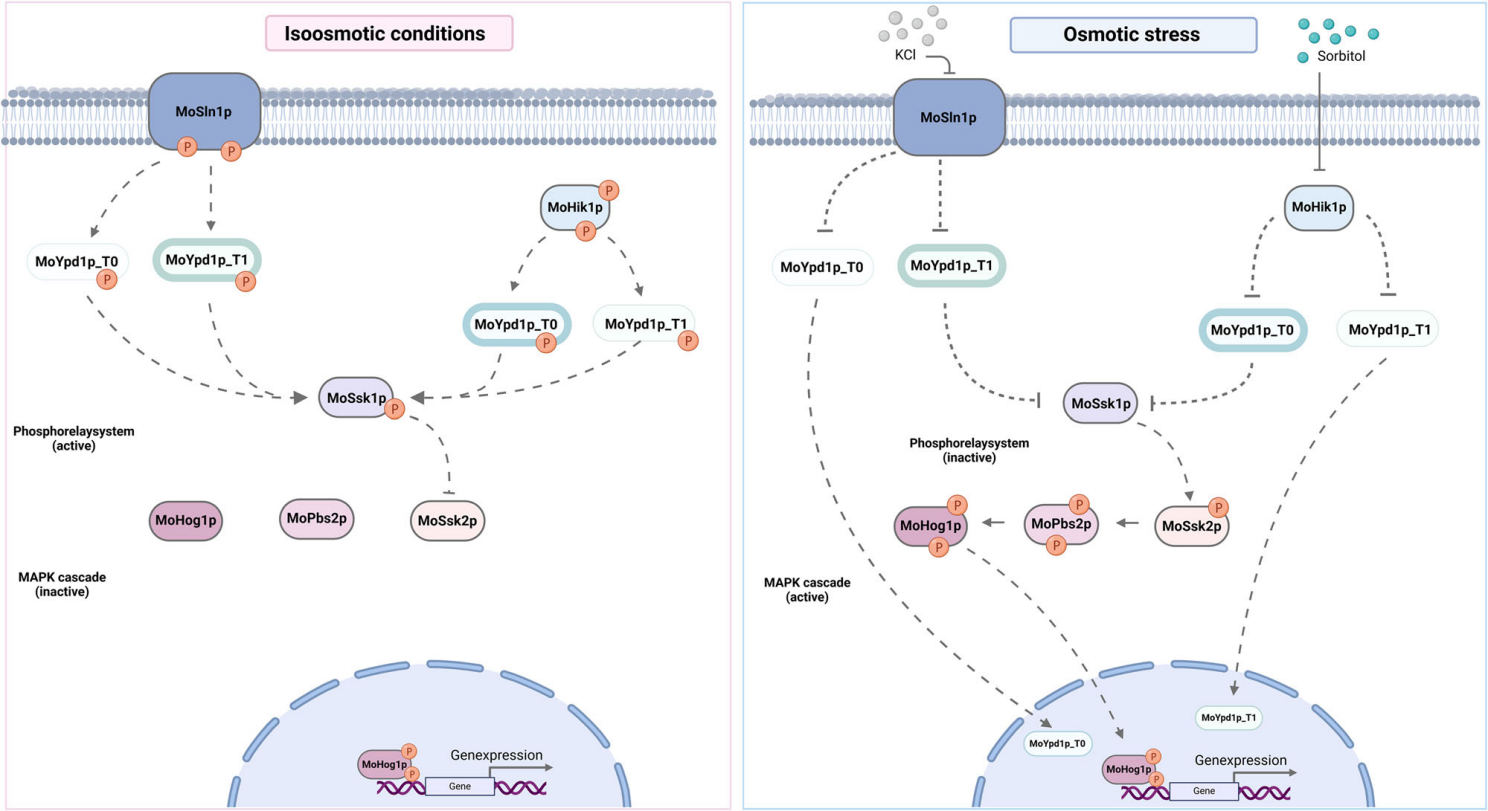
High osmolarity glycerol pathway in *M. oryzae*. MoYpd1p_T0 and MoYpd1p_T1 translocate to the nucleus after sensing KCl and sorbitol stress by MoSln1p and MoHik1p, respectively. Depending on the stress, phosphorylated Mohog1p and dephosphorylated Moypd1p_T0 or MoYpd1p_1 initiate a response in the nucleus.

Based on the results presented in this study, we concluded that the *MoYPD1_T0* isoform is dominantly expressed after KCl stress. By contrast, the *MoYPD1_T1* isoform was found to be expressed mainly after sorbitol stress. In accordance with our observations of nuclear translocations of the GFP-fused protein isoforms MoYpd1p_T0 and MoYpd1p_T1 upon KCl and sorbitol stress induction, respectively, we present an extension of the current model of the HOG pathway including these isoforms (Fig. 5). The phosphorelay system is active in isoosmotic (untreated) conditions, thereby inhibiting the downstream MAPK cascade. Phosphorylated MoYpd1p isoforms are in the cytoplasm. Following KCl and sorbitol stress perception by MoSln1p and MoHik1p, respectively, MoYpd1p_T0 and MoYpd1p_T1 translocate into the nucleus immediately after stress induction. High osmolarity inactivates the phosphorelay system, activates the MAPK cascade and leads to phosphorylation of MoHog1p, which is thereby translocated to the nucleus. According to our findings, MoHog1p and the isoforms MoYpd1p_T0/T1 or a combination of them are probably responsible for initiating the nuclear reaction.

## Discussion

Globally, *M. oryzae* is one of the most devastating agricultural pathogens^30,31^. That is down to the fact that rice blast disease is a major threat to the food security of approximately half of the world’s population who depend on rice as their primary food. A better understanding of the pathogen’s biology is essential to address this problem. Pathogenicity-related processes are shaped by differentiation, multicellular communication mechanisms, modulation or rewiring of cellular pathways, and/or suppression of defense mechanisms^32–34^. Consequently, the development of effective control strategies based on a deeper understanding of signaling mechanisms involved in fungal virulence is a promising path in plant protection^35,36^. *M. oryzae* needs to adapt rapidly to changing osmotic conditions in the plant tissue during the intracellular invasion of the host plant. Osmoregulation in fungi is coordinated via the HOG pathway^37^. Although the latter has been well studied in *Saccharomyces cerevisiae*, its regulatory network and contribution to the virulence of fungal pathogens remains to be elucidated^38,39^. The HOG pathway is also known as the target of the fungicide fludioxonil, however, its mode of action is not completely understood^18,40^. Based on yeast two-hybrid experiments, it was found that the phosphotransferase (HPt) MoYpd1p acts as the key mediator of the phosphorelay system of the HOG pathway, interacting with the two sensor HKs MoHik1p and MoSln1p as well as the RR MoSsk1p^24^. Signaling systems, such as phosphorelay systems, generally use proteins containing Hpt to transmit signals from sensor HKs to RRs^36,41^.

Based on RNA-Seq and HPLC-MS/MS analyses, we previously hypothesized that AS may produce several different MoYpd1p isoforms with distinct physiological functions^26,27^. Apart from MoSln1p and MoHik1p, MoYpd1p is likely to interact with one or more of the remaining eight HKs (MoHik2p-MoHik9p) identified in *M. oryzae*^19^. The aim of the presented investigation was to identify novel *MoYPD1* isoforms in silico and determine their role in pathogen signal-processing during infection in vivo. Up to now, the scientific understanding of AS and the role of different isoforms during fungal infection processes and development has still been limited^10,16^. However, numerous studies in plants have shown that AS is a crucial posttranscriptional mechanism for regulating gene expression and produces various isoforms, thereby, controlling biotic and abiotic stress responses^42–44^. The RGA5 resistance gene in rice (*Oryza sativa*), for example, is transcribed into the two isoforms RGA5-A and RGA5-B, conferring resistance of rice to *M. oryzae* effector proteins AVR-Pia and AVR1-CO39^45–47^. Previous studies in plants have also demonstrated that distinct Hpt proteins interact differently with RR, thereby, influencing signal transduction in the HOG and cytokinin signaling pathways^48,49^. Contrary to this, the effect of isoforms as virulence factors in the fungal kingdom is rare. In order to change and tackle this, we identified different isoforms of *MoYPD1* and elaborated their contribution to fungal physiology and pathogenicity in *M. oryzae*.

It is generally well accepted that signaling proteins containing Hpt, such as Ypd1p, are central constituents of pathogenicity-related differentiation and fungal virulence^50,51^. The first hint of the existence and involvement of multiple MoYpd1p isoforms in *M. oryzae* pathogenicity resulted from our comparison of disease symptoms on wounded rice leaves inoculated by different *Magnaporthe* mutant strains. In this study, plant assays with fungal mutants carrying single aberrant MoYpd1p isoforms highlighted, for the first time, the important role of each individual isoform acting together in a holistic machinery to obtain full virulence. Indeed, mutant strains producing just one sole MoYpd1p-isoform caused only a limited number of lesions on the rice leaves compared to the wildtype strain and the complemented mutant strain *ΔMoYpd1::MoYPD1-GFP*. These results are consistent with those of a transcriptome analysis of the *ΔMoYPD1* mutant of strain B157. Several genes involved in cell wall degradation and secondary metabolite production were found to be downregulated in this analysis^6^.

We present here the first report about the contribution of single isoforms of a signaling protein to pathogenicity-related processes in vivo in fungal pathogens. Moreover, these results are consistent with limited previous studies shedding light in the general linkage of AS and fungal virulence. Disease symptoms, for example, were detected on cabbage after inoculation of the wildtype strain of *Fusarium oxysporum f. sp. conglutinans (Foc*) and a mutant strain producing four isoforms of the effector protein SIX1. However, no disease symptoms were observed in the cabbage after inoculation of the *Foc-ΔSIX1* mutant strain^52^. In addition, infection studies on *Candia albicans* mutants that produce protein mannosyltransferase isoforms have shown that the virulence is dependent on not only the individual isoforms but also the environment in which they are infected^53^. The expression profiles of the yeast *Hortaea werneckii* highlight a specific role of the MAP2K HwPbs2 isoforms within the HOG pathway. Consequently, two HwPbs2 isoforms (HwPbs2A and HwPbs2B2) are involved in the rapid adaptation to a hypersaline environment, while a third isoform (HwPbs2B1) appears to respond to moderate salt stress^54^.

Identifying isoforms and elucidating their physiological functions require synergistic approaches of molecular biology and bioinformatics. Thereby, RNA-Seq data analysis as the only basis can be problematic for several reasons. Most published fungal RNA-Seq data are not strand-specific, and transcripts frequently overlap between adjacent genes due to their high gene density^55^. Furthermore, choosing between sequencing technologies and a wide range of bioinformatic tools that perform similar tasks but have different advantages and disadvantages can be challenging^56,57^. We initially identified various *MoYPD1* isoforms using both short-read Illumina RNA-Seq and long-read PacBio Iso-seq sequencing methods, and subsequently verified their existence at the cDNA level^8,58–61^. Based on the isoform expression analyses, putative signal-specific functions of the isoforms were investigated. The expression of isoform *MoYPD1_T0* increases after treatment with KCl, whereas the expression of isoform *MoYPD1_T1* increases after treatment with sorbitol (Fig. 4). Interestingly, it was found that fludioxonil increased strongly the expression of isoform T4 which was a different response as compared to KCl and sorbitol.

That indicates a putative role of MoYpd1p_T0 in salt stress response and MoYpd1p_T1 in sorbitol stress response. That is in line with the GFP studies, in which isoform MoYyp1p_T0 translocates into the nucleus after KCl stress, whereas MoYpd1p_T1 was found within the nucleus after sorbitol stress (Fig. 3). It was, therefore, hypothesized that the isoforms coexist and synergistically control stress responses and pathogenicity. Our hypothesis is supported by the transcriptomic analysis of field isolate KJ201, which detected infection-specific AS in about 500 genes^16^. However, in vivo studies on the biological function of isoforms of the pathogen are still needed. The role of MoB56 isoforms, for example, a regulatory subunit of protein phosphatase 2A, is not yet fully understood^62^.

To conclude, this study provides a new model of the HOG signaling pathway *in M. oryzae* and underpins the major role of signaling pathways in fungal pathogenicity by adding AS of signaling proteins as virulence determinants for the first time.

## Materials and methods

### Fungal growth condition and sampling

The *M. oryzae* wildtype 70-15 and the *ΔMoypd1* mutant strain were routinely maintained at 26 °C on complete medium (CM, pH 6.5, 2% agar, containing per liter: 10 g glucose, 1 g yeast extract, 2 g peptone, 1 g casamino acids, 50 ml nitrate salt solution (containing per liter: 120 g NaNO_3_, 10.4 g KCl, 30.4 g KH_2_PO_4_, 10.4 g MgSO_4_ × 7 H_2_O) and 1 ml of a trace element solution (containing per liter: 22 g ZnSO_4_ × 7 H_2_O, 11 g H_3_BO_3_, 5 g MnCl_2_ × 4 H_2_O, 5 g FeSO_4_ × 7 H_2_O, 1.7 g CoCl_2_ × 6 H_2_O, 1.6 g CuSO_4_ × 5 H_2_O, 1.5 g Na_2_MoO_4_ × 2 H_2_O, 50 g Na_2_EDTA, pH 6.5 adjusted by 1 M KOH). Regarding RNA isolation, conidia of 14-day-old *M. oryzae* cultures were harvested with H_2_O, filtered over two layers of Miracloth (Merck, Darmstadt, Germany), and used for the inoculation of 250 ml liquid CM (5 ×10^4^ conidia/ml) in a 500 ml glass flask. After three days of incubation at 26 °C and 120 rpm in a 12:12 light-dark cycle, an untreated sample was taken before the induction with stress (KCl [0.5 and 1 M], NaCl [0.75 M] sorbitol [0.5 and 1 M] and fludioxonil [10 μg/ml], respectively). Samples were then collected 25 min and 180 min after stress exposure, frozen in liquid nitrogen and stored at – 80 °C.

### Generation of *YPD1*-fused GFP vectors

**Fig. 6:**
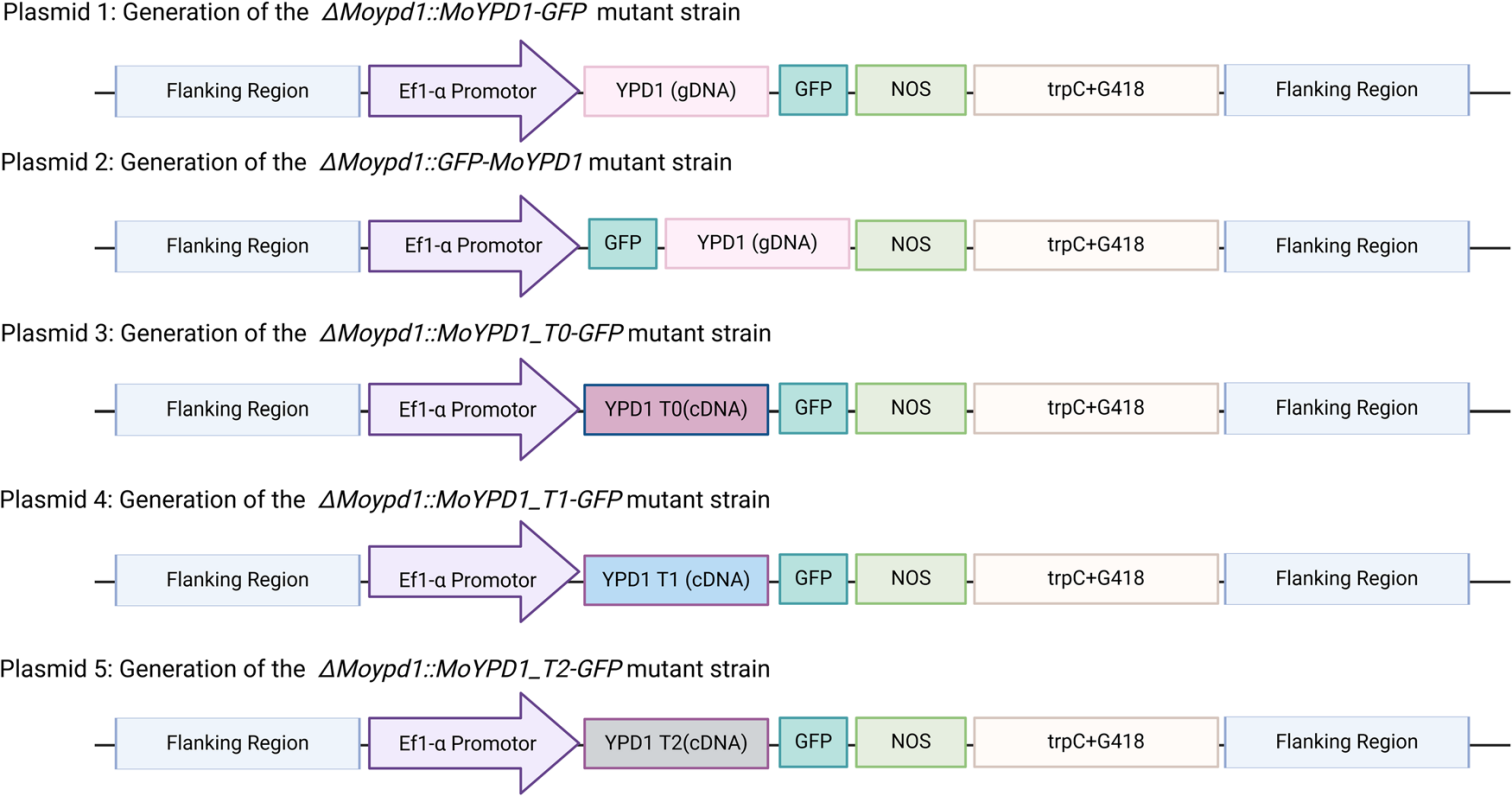
Overview plasmid design via Gibson Assembly. Schematic representation of the construction of the different plasmids is shown. The amplified fragments and their order are visualized in different colours.

The process of isolating genomic DNA and transforming plasmids into NEB^®^ 10-β competent *Escherichia coli* strains was carried out as described previously^27^. Detailed information on the plasmid design and the primers required for the Gibson Assembly^®^, designed using the NEBuilder tool (https://nebuilder.neb.com/#!/), are provided in Fig. 6 and Supplementary Table S1. MoYpd1p localization was investigated by mutants being transformed with plasmids in which either N-terminal or C-terminal GFP sequences have been fused to the genomic sequence of “total” *MoYPD1* (locus MGG_07173) or only to the CDS of the isoforms *MoYPD1*_T0 (MGG_07173T0) or *MoYPD1*_T1 (MGG_07173T1), as well as the recently discovered *MoYPD1*_T2^27^. Total *MoYPD1*, 3’- and 5’-flanking sequences as well as the *Ef1*α promotor (of the gene MGG_03641, elongation factor 1 alpha) were amplified from gDNA whereas the single isoform-specific plasmids were generated by using cDNA as template. The remaining fragments were obtained from the *Agrobacterium*-compatible binary plasmid *pSJ* + *GFP*(G418)^25^, which served as the backbone for Gibson Assembly® after digestion with *Xho*I and *Bgl*II. The seven fragments of each plasmid 1–3 were assembled in one single reaction (Fig. 6). Regarding the construction of plasmids 4 and 5, plasmid 3 was restricted with *XhoI* and *Bgl*II in order to introduce the respective isoform into the resulting backbone. The correct assembly was always confirmed by Sanger sequencing. Fungal transformation of the *M. oryzae* wildtype and the *ΔMoypd1* mutant strain was conducted using *Agrobacterium tumefaciens*-mediated transformation^25^. The geneticin resistance cassette (G418) and the hygromycin resistance were used as resistance marker genes.

### Microscopy

Fluorescence microscopy of the GFP-fused *MoYPD1* strains was performed on the Revolution microscope (Echo) using Revolution software version 108.1. Conidia from 14-day-old fungal cultures grown on culture media agar plates were filtered through two layers of Miracloth (Merck) and incubated with Hoechst 33342 solution (20 mM, Thermo Fisher Scientific™) for 30 min at room temperature. The conidia were then mixed 1:1 with Biozym Low Melt Agarose (1.0% w/v) and transferred to the 8-well chambers (ibidi ^®^). The NaCl [0.75 M], KCl [0.75 M and 1 M] or sorbitol [1 M] were added as stress-inducing agents.

### Plant infection assay

Conidia of the wildtype strain 70-15, *ΔMoypd1* and the respective *MoYpd1p* isoform-producing strains were harvested from 14-day-old cultures in order to assess virulence. The plant infection assays were carried out using 21 day old plants of dwarf indica rice cultivar CO-39. Plants were cultivated using a daily cycle of 16 h light followed by 8 h darkness (28 °C, 90% relative humidity). Conidial suspensions were adjusted to 5 × 10^4^ conidia/mL in H_2_O containing 0.2% gelatin. Five rice plants were spray-inoculated with each 10 mL of conidial suspension and were incubated in plastic bags. After 5 days of incubation, lesions were counted.

### Vegetative growth and germination assay

Mutant strains were tested for stress tolerance on agar plates. Therefore, agar blocks of 0.5 cm diameter were cut from the outer growth zone of the cultures to be tested and placed onto freshly prepared CM or minimal medium agar plates (MM, pH 6.5, 2% agar, contains per litre: 1 g glucose, 50 mL nitrate salt solution, 0.25 mL biotin-solution (0.01%), 1 mL thiamindichloride solution (1%) and 1 mL of a trace element solution) with different stress inducing compounds. The cultures were grown for 10 days at 26 °C and the colony diameter was measured. In addition, germination was investigated with conidia harvested from 11 day old *M. oryzae* cultures and the mutant strains, grown on complete medium (CM). The conidia were filtered through two layers of miracloth tissue to give a conidial suspension, which was adjusted to 5 × 10^4^ conidia/mL in H_2_O. Then, the stress inducing compounds were added and the samples were incubated at 26 °C for at least 16 h. The germination respectively the subsequent initial vegetative growth phase was monitored under the microscope.

### RNA Isolation and cDNA synthesis

The lyophilized mycelium was mechanically disrupted using the TissueLyserII system (QIAGEN, Hilden, Germany) for 30 s at 30 Hz. Total RNA was extracted using the RNeasy^®^ Plant Mini Kit, including RNase-free DNase treatment, according to the manufacturer’s instructions. Three biological replicates of each condition were pooled together. The RNA integrity numbers were evaluated by an Agilent 2100 bioanalyzer (Agilent Technologies, Santa Clara, CA, USA) employing the RNA 6000 Nano Kit (Agilent Technologies). Samples with RNA integrity number values above nine was selected for transcriptome analysis.

In order to detect new AS events by PCR and Sanger sequencing and plasmid construction, cDNA was synthesized with oligo(dT)20 primers with Invitrogen’s SuperScript^®^ III First-Strand Synthesis System for RT-PCR (Invitrogen, Germany), according to the manufacturer’s instructions. The cDNA used for ddPCR was transcribed using the iScriptTM Advanced cDNA Synthesis Kit for RT-qPCR (Biorad, Munich, Germany).

### PCR and ddPCR

Primer design (https://www.ncbi.nlm.nih.gov/tools/primer-blast/) was based on the results of Cufflinks, MAJIQ and SGSeq. The PCR was carried out in a total volume of 25 µL containing 1.25 µL of each primer [100 pmol/µL], 0.5 µL of dNTPs [10 mM], 2 µL of cDNA [100–600 ng/µl], 5 µL of 5 × Q5^®^ Reaction buffer, 0.5 µL of Q5^®^ DNA polymerase (NEB, Frankfurt, Germany), and 14.5 µL of RNase-free water to perform the reaction. Primer-specific annealing temperatures (Tm) were determined in advance using NEB Tm Calculator version 1.15.0, and extension times were calculated according to the manufacturer’s instructions. The PCR was carried out in a C1000 Touch Thermal Cycler (BioRad Laboratories, Hercules, CA, USA) under the following amplification parameters: initial denaturation for 30 s at 98 °C, 35 cycles consisting of denaturation for 10 s at 95 °C, annealing corresponding to primer-specific tm for 15 s, extension at 72 °C for the calculated extension time, and a final extension step for 5 min at 72 °C. The PCR product was separated by electrophoresis in 1% agarose stained with ethidium bromide, and visualized using a QUANTUM-ST5-1100/26MX system (PEQLAB Biotechnologie GmbH, Erlangen, Germany). Amplicon gel extraction following the Monarch^®^ DNA Gel Extraction Kit (NEB, Frankfurt, Germany) instructions was used to isolate and purify the cDNA. Next, the GeneJET Plasmid Miniprep Kit (Thermo Fisher Scientific GmbH, Schwerte, Germany) was used to purify the plasmid DNA after cloning the PCR products using the CloneJET™ PCR Cloning Kit with DH10B Competent Cells (Thermo Fisher Scientific GmbH, Schwerte, Germany). Sanger sequencing was performed (Eurofins Genomics, Germany) to determine the sequence similarity between the amplified cDNA sequence and the *MoYPD1* sequence, followed by BLASTN analysis (https://blast.ncbi.nlm.nih.gov/Blast.cgi) and the prediction of ORFs (www.ncbi.nlm.nih.gov/orffinder).

We used the cDNA as a template for the ddPCR for the absolute quantification of a novel AS-Event. This technique is based on water emulsion droplet technology and fractionates the target template into 20,000 droplets. As a result, the PCR amplification of the template is carried out in each droplet, and counting the positive droplets allows for a precise, absolute determination of the target’s concentration. The ddPCR was performed using three independent replicates. A ddPCR reaction mixture of 11 μl QX200 EvaGreen Supermix (Bio-Rad Laboratories), 1.1 µl of each primer [2 pmol/µl], 1 µl cDNA [1000 ng/µl] and 7.8 µl water was used for the droplet formation. After droplet generation using the QX200 Droplet Generator (Bio-Rad Laboratories), 40 μl of sample was transferred to a 96-well plate (Bio-Rad Laboratories), which was heat-sealed at 180 °C for 5 s before amplification. The PCR was started by enzyme activation at 95 °C for 5 min, followed by 40 cycles of amplification at 95 °C for 30 s (denaturation) and 60 °C for 1 min (annealing/extension). After signal stabilization at 4 °C for 5 min and 90 °C for 5 min with a temperature ramp of 2.5 °C/s, the 96-well plate was incubated overnight at 12 °C. On the next day, an analysis was carried out using a QX2000 Droplet Reader (manufactured by Bio-Rad Laboratories). The reader utilizes Quanta Soft software, 1.2 standard edition (also manufactured by Bio-Rad Laboratories) in conjunction with a two-color detection system to analyze each droplet.

### Sequencing

Illumina and PacBio Iso-Seq sequencing systems generated the RNA sequencing data in this study. Library preparation and Illumina 100 bp paired-end sequencing of total RNA were performed by Eurofins Genomic Europe Sequencing GmbH (Germany), followed by a raw data quality assessment with QualiMap version 2.2. (https://github.com/EagleGenomics-cookbooks/QualiMap). Depending on the analysis tool, TopHAT (Galaxy version 2.1.1) and HISAT2 (Galaxy version 2.2.1+ galaxy1) aligned the raw FASTQ data derived by short-read sequencing along the 70-15 reference strain genome (assembly version MG8, http://fungi.ensembl.org/Magnaporthe_oryzae/Info/Index). Furthermore, full-length transcript analysis was performed using PacBio’s Iso-Seq method at the Berlin Institute of Health at Charité (Germany). After Iso-Seq library preparation and sequencing on the PacBio Sequel II platform (one SMRT^®^ Cell 8 M), raw reads were classified into circular consensus sequences and non- circular consensus sequences subreads and processed as described in IsoSeq3 pipeline version 3.8.1 (https://isoseq.how/). To this end, the Pigeon workflow was used for mapping, merging and classifying transcripts according to the IsoSeq3 cluster of the bulk workflow.

### Alternative splicing analysis of *MoYPD1*

Several approaches, including isoform-based (Cufflinks/Cuffdiff) and splicing event-based (rMATS, MAJIQ, and SGSeq) analysis, were conducted for the prediction of *MoYPD1* isoforms. The expression values were determined using StringTie and Ballgown and DESeq2.

### Cufflinks

**Fig. 7:**
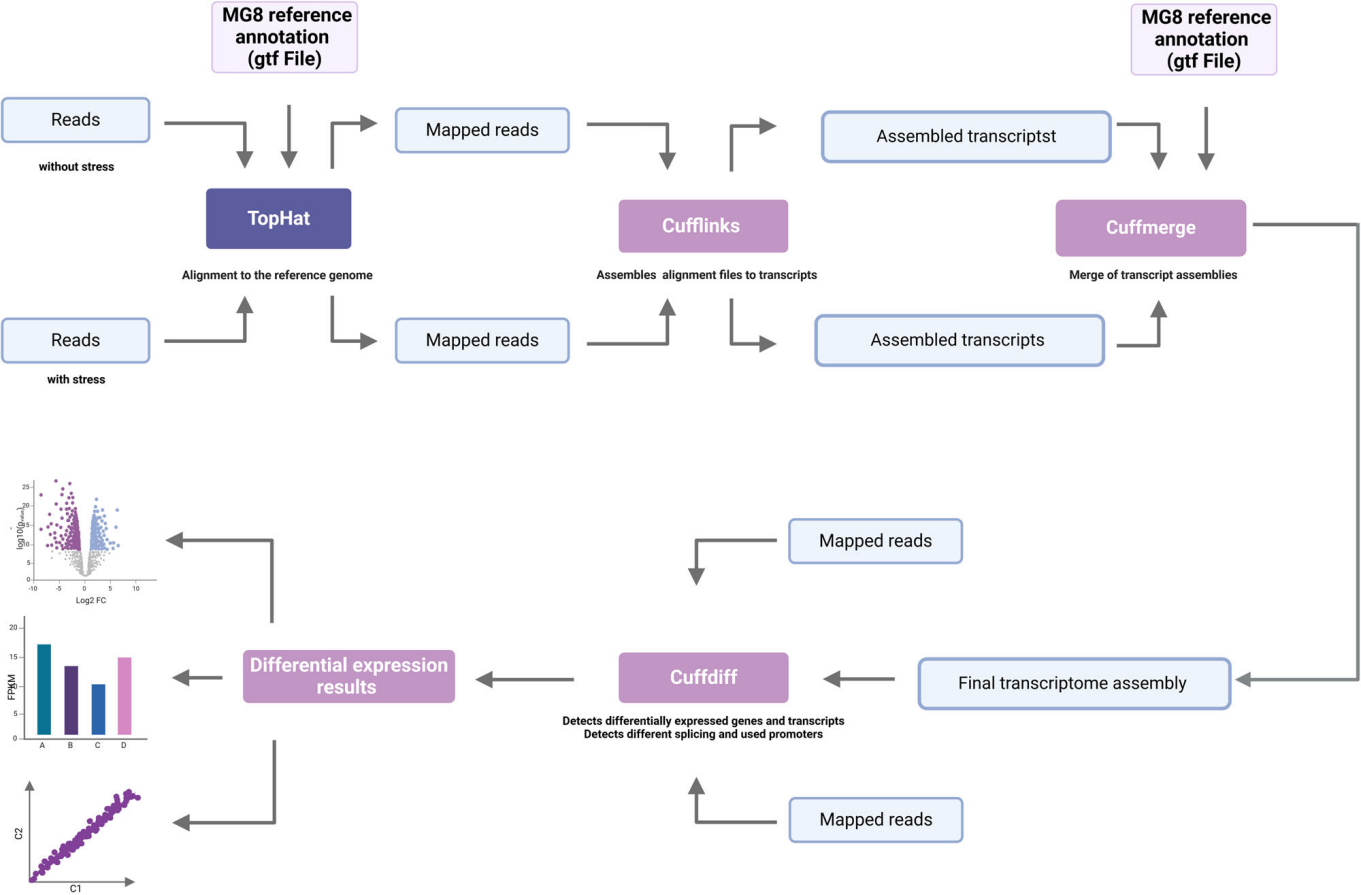
Workflow of RNA Seq analysis via Cufflinks. The order of different bioinformatic workflows is schematically represented. Cufflinks was used, then FASTQ files of two experimental conditions (with and without stress induction) were mapped to MG8 reference annotation using TopHat. Based on the resulting SAM or BAM file, Cufflinks assembled the alignment files independently of the reference annotation to possible transcripts and generates transcriptome assemblies for each condition. The assemblies of each condition and the MG8 reference annotation were used for merging by Cuffmerge, resulting in a final transcriptome assembly. Following this, TOPHAT-based BAM files and the final transcriptome assembly were used for isoform-based analysis with Cuffdiff. The R package was used for visualization.

Cufflinks, as an isoform-based method, involves several programs, including Cufflinks itself, Cuffmerge and Cuffdiff (Fig. 7). Cufflinks version 2.2.1 was used, following the instructions available at http://cole-trapnell-lab.github.io/cufflinks/manual/. FASTQ files of two experimental conditions (with and without stress induction) were mapped to MG8 reference annotation using TopHat. Based on the resulting SAM or BAM file, Cufflinks assembled the alignment files independently of the reference annotation to possible transcripts and generates transcriptome assemblies for each condition (GTF files). The assemblies of each condition and the MG8 reference annotation were used for merging by Cuffmerge, resulting in a final transcriptome assembly (GTF file). Following this, TOPHAT-based BAM files and the final transcriptome assembly (GTF file) were used for isoform-based analysis with Cuffdiff. Finally, the R package cummerbund version 2.38.0 was used for visualization; it reads the output files from Cuffidff into an S4 object, making the data easily accessible for graphical representation.

### rMATS, MAJIQ and SGSeq

RNA-Seq data were also analyzed with HISAT2-produced alignments and count-based methods. The MG8 annotations were processed to generate AS events using rMATS turbo version 4.2.1, as described at https://github.com/Xinglab/rmats-turbo/blob/v4.1.2/README.md. Data were filtered with a cut-off *p*-Value of 0.05. In contrast to Cufflinks, rMATS provides explicit AS events rather than whole transcripts. The identification of known and de novo splice connections were performed using SG-Seq and MAJIQ. Local splicing variations were analyzed with MAJIQ version 2.4, conducted as described at https://biociphers.bitbucket.io/majiq-docs-academic/. Local splicing variations can be visualized as splits in splice graphs where several edges are connected to or derived from a single exon, called the reference exon^61^. Based on the aligned reads, the MAJIQ Builder constructed a splice graph for *MoYPD1*, then local splicing variations for each condition are quantified and compared with the MAJIQ Quantifier, calculating the percent inclusion index (Ψ) and its change (ΔΨ). Finally, the Viola visualization package displayed the results of the MAJIQ Builder and Quantifier as a splice diagram with alternative splice variants and violin plots of *MoYPD1* depicting the Ψ and ΔΨ estimates. The AS events were also identified with SGseq version 1.30.0, an R/Bioconductor package (https://bioconductor.org/packages/release/bioc/vignettes/SGSeq/inst/doc/SGSeq.html). Mapped reads are used to predict splice junctions and exons in a genome-wide splice graph. Based on reads extending across the start or end of each splice variant, recursive splice events are identified on the graph and quantified locally^58^.

### Sashimi plot

The visualization of splice junctions from HISAT2-aligned RNA-seq data along the *MoYPD1* gene of reference strain 70-15 was performed as described at https://github.com/guigolab/ggsashimi. Arcs representing introns include the number of reads spanning an exon in Sashimi plots. Only splice junctions with a minimum read coverage of 15 for *MoYPD1* are shown.

### StringTie and Ballgown

Heat plots for transcript expression were created using StringTie version 2.2.1 and Ballgown version 2.28.0. StringTie, firstly, builds AS graphs and then performs a heuristic algorithm to determine the heaviest path, which represents a transcript. Afterwards, a flow network design is used to determine the coverage of each transcript^63^. Here, the Ballgown compatible output was generated providing a HISAT2-generated alignment and the MG8 annotation (GTF file) for StringTie, and was used subsequently to visualized the transcripts and their expression level under different conditions, as described at https://github.com/alyssafrazee/ballgown.

#### DeSEq2

Salmon version 1.9.0 (https://salmon.readthedocs.io/en/latest/) was used for the initial quantification of transcripts. The differential expression analysis was conducted using version 1.36.0 of DESeq2 (Differential Expression Analysis for Sequencing Data 2), according to http://www.bioconductor.org/packages/release/bioc/vignettes/DESeq2/inst/doc/DESeq2.html.

### Reporting summary

Further information on research design is available in the Nature Portfolio Reporting Summary linked to this article.

### Supplementary information

Supplementary Information

Description of Additional Supplementary Files

Supplementary Data 1

Reporting Summary

## Data Availability

For source data of Fig. 3 and Fig. 4, all raw data used in this study is public available on figshare repository (10.6084/m9.figshare.24763470.v1). For the raw data of the graphs in Figure S3, see the supplementary data 1.
