# Supplementary material for: An array of signal-specific MoYpd1 isoforms determines full virulence in the pathogenic fungus Magnaporthe oryzae
Source: Commun Biol. 2024 Mar 4;7:265. doi: 10.1038/s42003-024-05941-z (PMC10912366; doi:10.1038/s42003-024-05941-z)
Supplement: Supplementary file 1 — Supplementary Information [file 42003_2024_5941_MOESM1_ESM.pdf]

Supplementary data

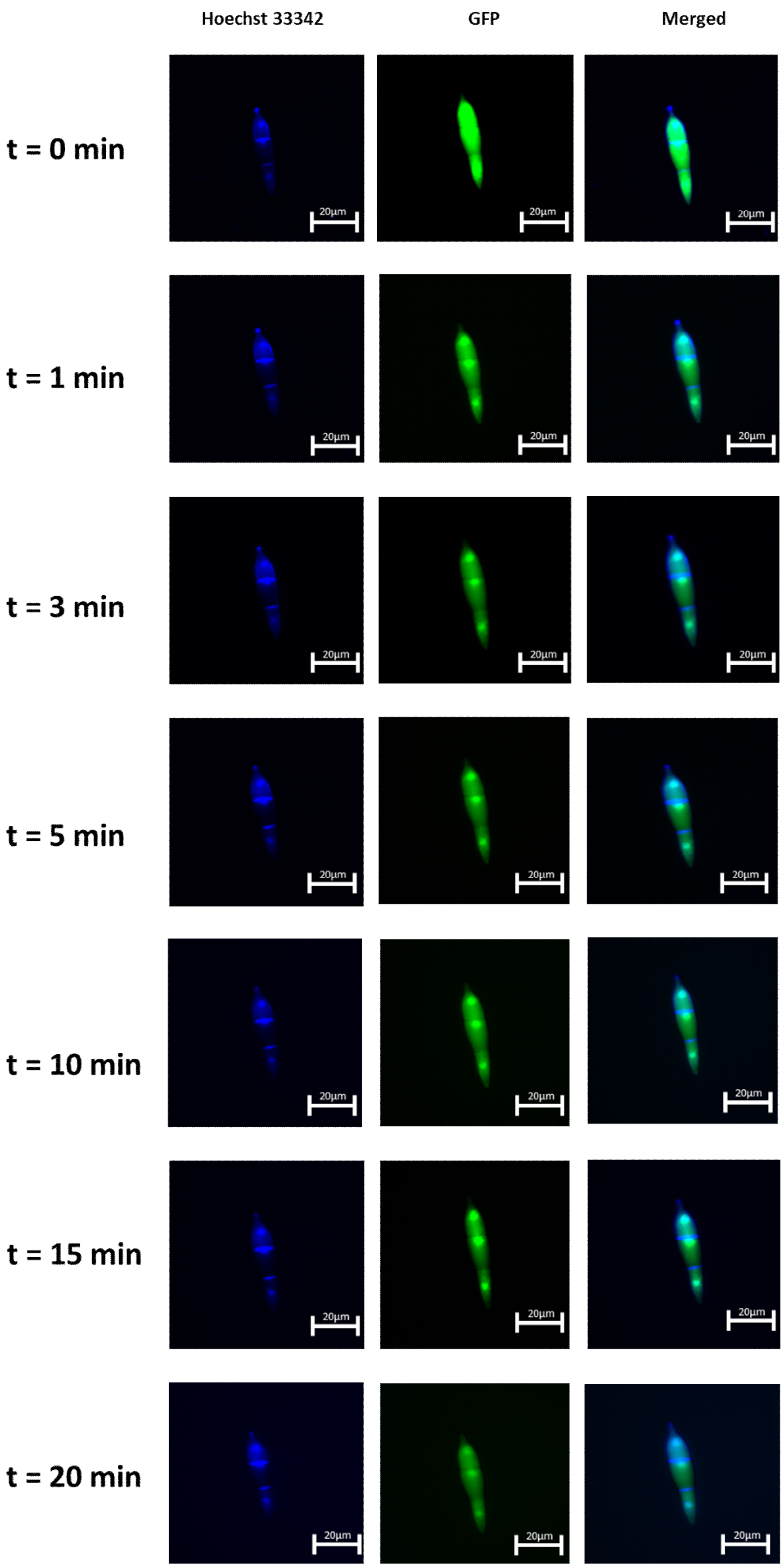

Figure S1: **Localization of GFP-fused total MoYpd1p in conidia of *ΔMoypd1::GFP-MoYPD1* before and after 1 M KCl treatment.**

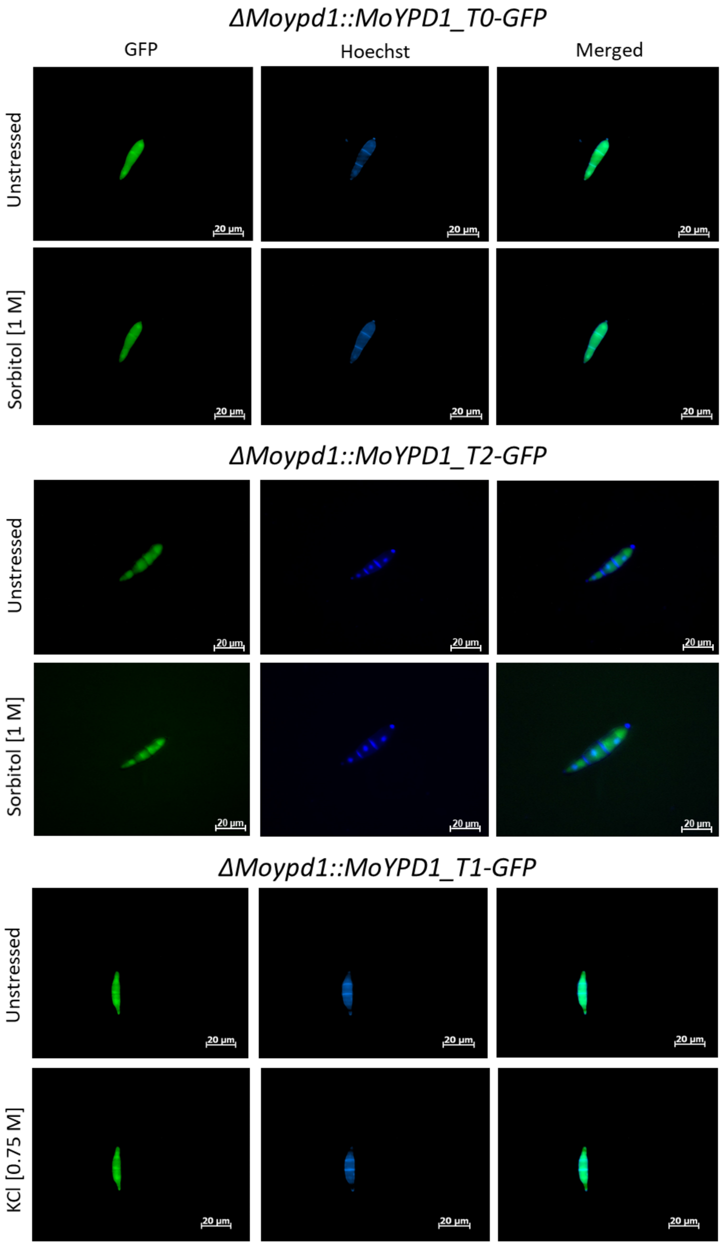

Figure S2: Localization of GFP-fused MoYpd1p isoforms. After treatment with sorbitol [1 M], no translocation into the nucleus was observed for MoYpd1p_T0 and MoYpd1p_T2. However, MoYpd1p_T1 did not accumulate in the nucleus upon KCl stress [0,75 M].

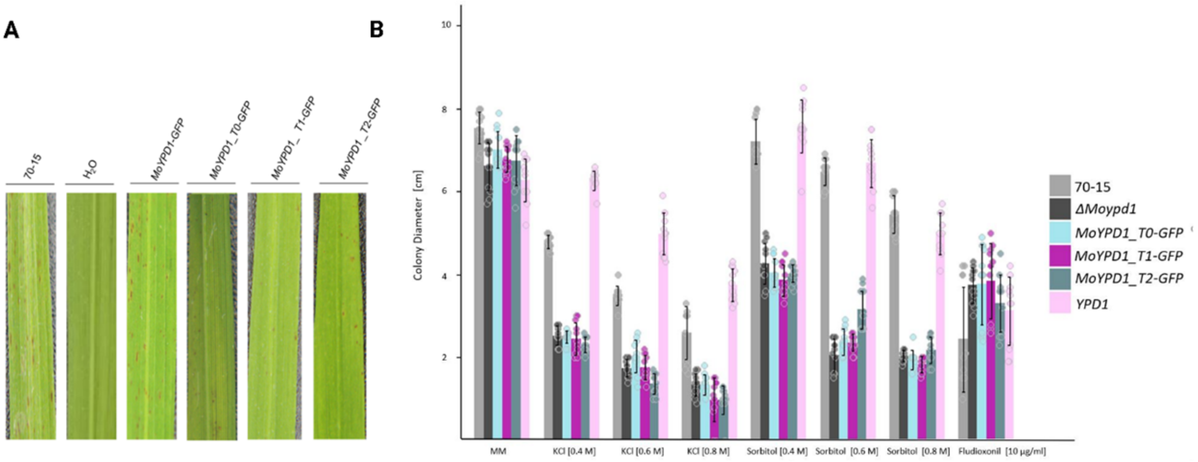

Figure S3: A: Rice leafs with blast lesions. Rice plants inoculated with conidia suspension of the mutant strains producing only one isoform show disease symptoms on the leaves. B: Vegetative growth of *Magnaporthe oryzae* 70-15 strain, the loss-of-function mutant Δ*Moypd1* and the GFP-tagged MoYpd1p-producing isoform strains under KCl, sorbitol and fludioxonil stress.

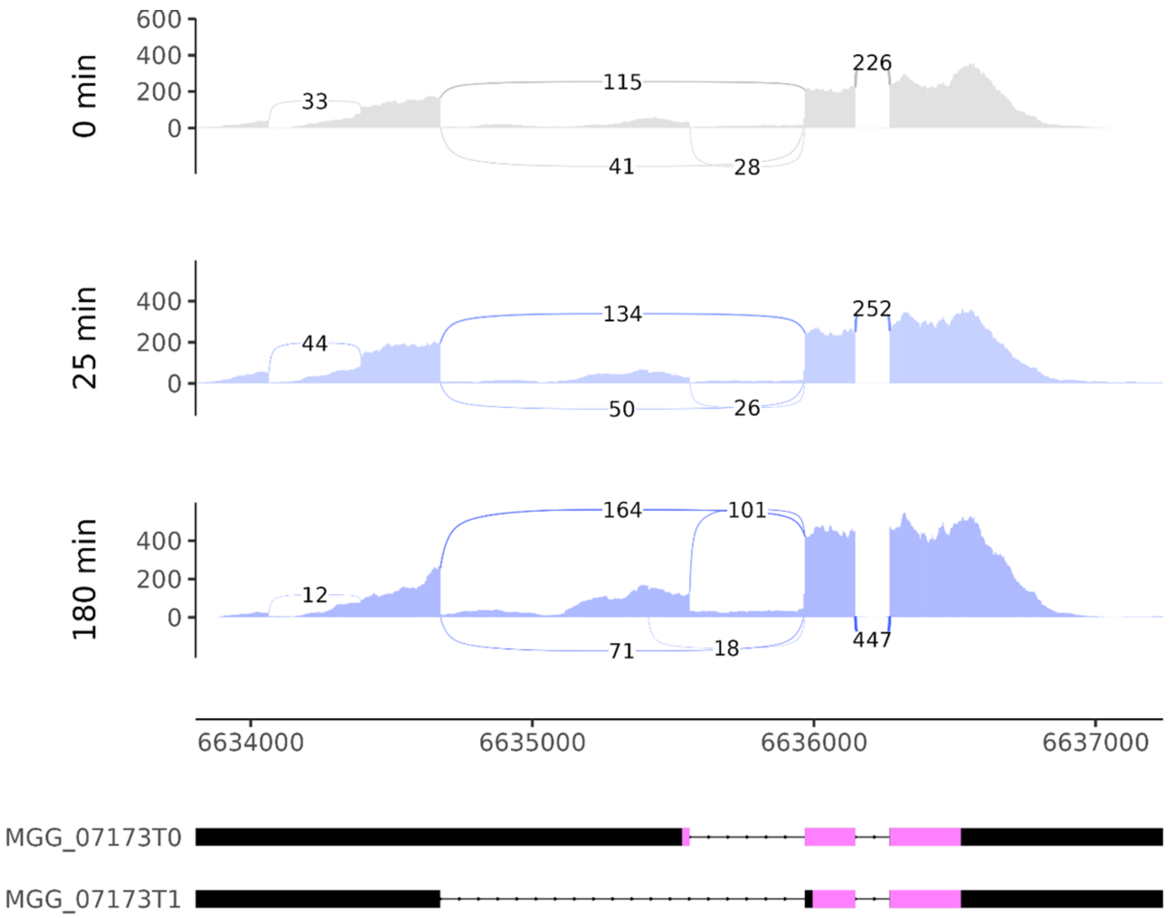

Figure S4: **Sashimi plots of *MoYPD1* before and after 0.5 M sorbitol treatment.** Sashimi plots visualize the read coverage (y-axis) for corresponding MoYPD1 genomic region (x-axis). Arcs represent splice junctions of *MoYPD1*. Beneath the sashimi plot annotated transcripts are shown with the CDS highlighted in pink.

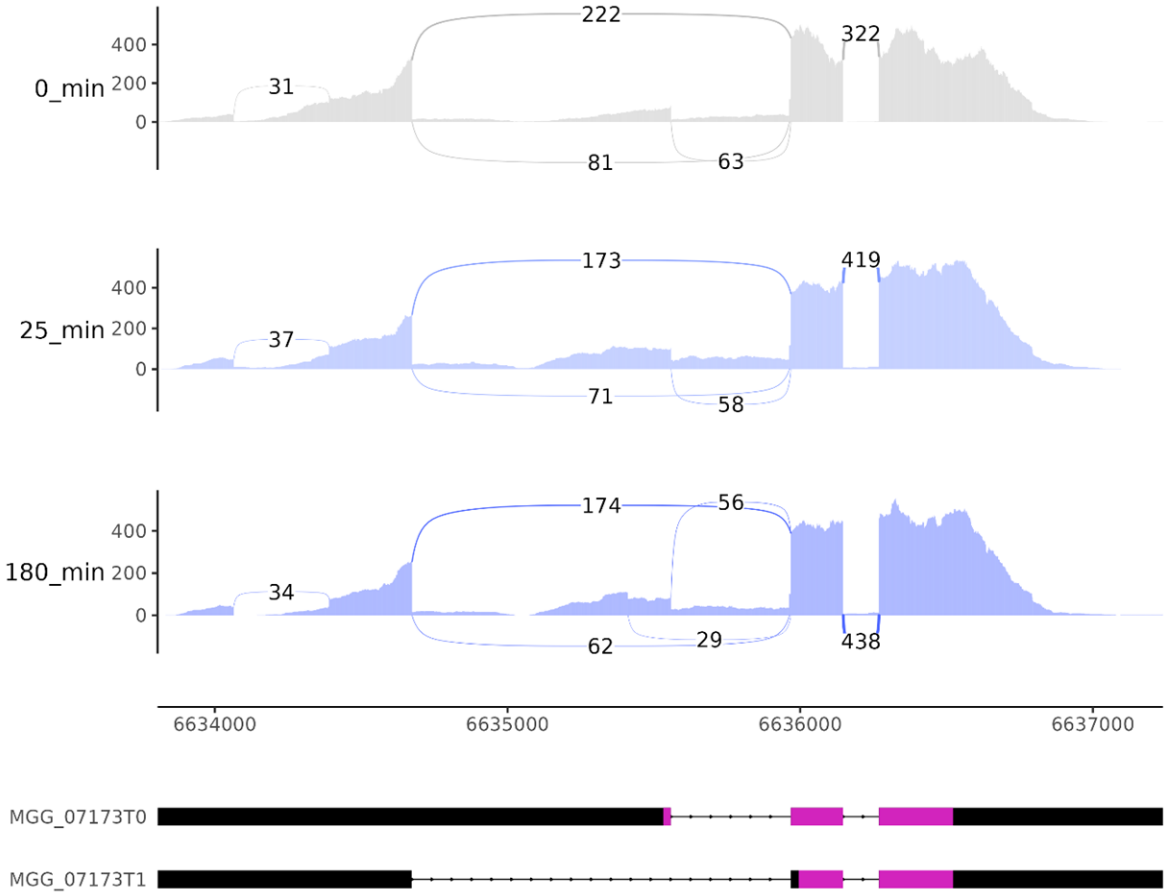

Figure S5: **Sashimi plots of *MoYPD1* before and after 10 µg/ml fludioxonil treatment.** Sashimi plots visualize the read coverage (y-axis) for corresponding MoYPD1 genomic region (x-axis). Arcs represent splice junctions of *MoYPD1*. Beneath the sashimi plot annotated transcripts are shown with the CDS highlighted in pink.

Table S1: **Primer Gibson Assembly®**

Primer ID	Oligonucleotide sequences (5'→3')
Plasmid 1	
SJ1561	ttactgatcactgattaagtactgatatcggtacgaaaaaaaaacccccttcaaagccctc
SJ1562	ttttttctcgctctcagcaaacaaccgtaaagatgtgagttc
SJ1563	atctttacggttgtttgctgagagcgagaaaaaaaaactcttcgtc
SJ1564	ctcctcctcctccgacatggtggcggtttggtgctctcttttt
SJ1565	agcaccaaaccgccaccatgtcggaggaggaggaggagaaca
SJ1566	tcctcgcccttgctcacaggatcgatcacgaaaaactcattgagaagtttc
SJ1567	tttttcgtgatcgatcctgtgagcaagggcgaggagctgttca
SJ1568	tgctccttcaatatcaggatctagtaacatagatgacaccgcgcgc
SJ1569	catctatgttactagatcctgatattgaaggagcattttttgggc
SJ1570	gtttgaagtttcttgaattcagaagaactcgtcaagaaggcgatag
SJ1571	ttgacgagttcttctgaattcaagaaacttcaaacgaaagac
SJ1572	cgaagaactcctccgcttcaagtcaagaatgcgcaacgctgaagggtatgaatcaataatg
Plasmid 2	
SJ1714	taagtactgatatcggtacgCTCGAGCTTGCTGGCTGTG

SJ1715	tcgctctcagTCGTGTACGCTCTGCTAGC
SJ1716	gcgtacacgaCTGAGAGCGAGAAAAAAAAC
SJ1717	acgcggccgcCTTGTACAGCTCGTCCATG
SJ1718	gctgtacaagGCGGCCGCGTACTCTGTAAAG
SJ1719	gatcgaattcCGCGGCCGCCTAAGGATC
SJ1720	ggcggccgcgGAATTCGATCGTTCAAAC
SJ1721	ttgaatctaaGAATTCTCAGAAGAACTCG
SJ1722	ctgagaattcTTAGATTCAAGAAACTTCAAAC
SJ1723	cttcaagtcaagaatgcgcaCTGAAGGGTATGAATCAATAATG
Plasmid 3	
SJ2359	ttactgatcactgattaagtactgatatcggtacgTTTAGAATGATAATGCTTTGTCAGCCACC
SJ2360	ttttttctcgctctcagTTCCTTGTTCTTGGCGGTATGCTCA
SJ2361	accgccaagaacaaggaaCTGAGAGCGAGAAAAAAAAACTCTTCGTC
SJ2362	gtcctttacagagtacatctcgagTGGCGGTTTGGTGCTCTCTTTTTGA
SJ2363	ccaaaccgccactcgagATGTACTCTGTAAAGGACTCTGAAAAG
SJ2364	cccttgctcacagatctAGGATCGATCACGAAAAACTCATTG
SJ2365	tttttcgtgatcgatcctagatctGTGAGCAAGGGCGAGGAGCTGTTCA
SJ2366	atcaataatgacttgctcCATCATGCAACATGCATGTACTGTCTGATGTATTAAGAGTATAG
SJ2367	atgcatgttgcatgatgGAGCAAGTCATTATTGATTCATACC
SJ2368	cgaagaactcctccgcttcaagtcaagaatgcgcaTAGTCGACAATTAATCATCTGTGCTAG
Plasmid 4	
SJ2369	atcatcaaaaagagagcaccaaaccgccacc*ccctcgag*ATGCCCGATTTTGGAGCCCA
SJ2370	ccggtgaacagctcctcgcccttgctcacaagatctttAGGATCGATCACGAAAAACTCATTGAG
Plasmid 5	
SJ2371	caaaaagagagcaccaaaccgccacccctcgagATGTCGGAGGAGGAGGAGGAGAAC
SJ2372	gaacagctcctcgcccttgctcacaagatctttAGGATCGATCACGAAAAACTCATTGAGAAG

Table S2: **Overview of the predicted proteins with the UniprotID and the length of the assumed amino acid sequence (n.a. indicates not available)**

Protein	Uniprot ID	Length (AA)
MoYpd1p_T0	G4MTK9	153
MoYpd1p_T1	G4MTL0	135
MoYpd1p_T2	n.a.	162

Table S3: **cDNA sequences of isoformT0, T1 and T2.**

**Isoform T0 cDNA sequence (5'→3')**
ATGTACTCTGTAAAGGACTCTGAAAAGTCCGATTCCGAAGAAAACGCCGACAAGATGCCCGATTTTGGAG CCCATGTCGACAGCACGACGTTCGAACAGATCCTGGAAATGGACGAAGATGAGGCCGAAAGGGATTTCA GCAAACCTTTAGTCATGGGATTCTTTGAACAAGCAGAGGAGACTTTTGAAAAAATGGACAAGGCCCTGAAA GATCGTGACTTGAAAGAGCTTTCGAGCCTTGGCCACTTCCTCAAGGGTTCATCCGCCACTCTGGGCTTTA CAAAGGTCAAGGACAGCTGCCAAGTGATTCAACAGTACGGAAACAAGCTGAAACTAGACGGCACTGAGGA GCCAAGCGAGGATGTGTGCTATGAGAAGATCGATAAAGCACTTGTGGATGCCAAGAAGGATATGGAGAGT TTGAAGAAACTTCTCAATGAGTTTTTCGTGATCGATCCTTAG
**Isoform T1 cDNA sequence (5'→3')**
ATGCCCGATTTTGGAGCCCATGTCGACAGCACGACGTTCGAACAGATCCTGGAAATGGACGAAGATGAG GCCGAAAGGGATTTCAGCAAACCTTTAGTCATGGGATTCTTTGAACAAGCAGAGGAGACTTTTGAAAAAAT GGACAAGGCCCTGAAAGATCGTGACTTGAAAGAGCTTTCGAGCCTTGGCCACTTCCTCAAGGGTTCATCC GCCACTCTGGGCTTTACAAAGGTCAAGGACAGCTGCCAAGTGATTCAACAGTACGGAAACAAGCTGAAAC TAGACGGCACTGAGGAGCCAAGCGAGGATGTGTGCTATGAGAAGATCGATAAAGCACTTGTGGATGCCA AGAAGGATATGGAGAGTTTGAAGAAACTTCTCAATGAGTTTTTCGTGATCGATCCTTAG
**Isoform T2 cDNA sequence (5'→3')**
ATGTCGGAGGAGGAGGAGGAGAACAAGAAGACCAAAGTCGTGGGTCGGTGTGAATCCGATTC CGAAGAAAACGCCGACAAGATGCCCGATTTTGGAGCCCATGTCGACAGCACGACGTTCGAAC AGATCCTGGAAATGGACGAAGATGAGGCCGAAAGGGATTTCAGCAAACCTTTAGTCATGGGAT

TCTTTGAACAAGCAGAGGAGACTTTTGAAAAAATGGACAAGGCCCTGAAAGATCGTGACTTGAA
AGAGCTTTCGAGCCTTGGCCACTTCCTCAAGGGTTCATCCGCCACTCTGGGCTTTACAAAGGT
CAAGGACAGCTGCCAAGTGATTCAACAGTACGGAAACAAGCTGAAACTAGACGGCACTGAGGA
GCCAAGCGAGGATGTGTGCTATGAGAAGATCGATAAAGCACTTGTGGATGCCAAGAAGGATAT
GGAGAGTTTGAAGAAACTTCTCAATGAGTTTTTCGTGATCGATCCTTAG

Table S4: Overview of the total AS events in *M. oryzae* via rMATS

Sample	Treatment	5'ASS	3'ASS	SE	IR	MXE	Total
t0	-	1030	1290	434	689	31	3474
t25	KCl [0.5 M]	1129	1415	591	670	46	3851
t180	KCl [0.5 M]	1089	1430	553	697	43	3812
t0	-	1151	1299	464	717	30	3661
t25	sorbitol [0.5 M]	1015	1219	424	612	22	3292
t180	sorbitol [0.5 M]	1012	1281	432	617	25	3367
t0	-	1030	1290	434	689	31	3474
t25	fludioxonil [10 µg/ml]	852	1106	306	490	12	2766
t180	fludioxonil [10 µg/ml]	944	1225	328	621	28	3146

Table S5: ddPCR Primer

Primer ID	Oligonucleotide sequences (5'→3')
SJ2494	GACGTTCGAACAGATCCT
SJ2596	CTCGAAAGCTCTTTCAAGTC
