# Supplementary material for: An array of signal-specific MoYpd1 isoforms determines full virulence in the pathogenic fungus Magnaporthe oryzae
Source: Commun Biol. 2024 Mar 4;7:265. doi: 10.1038/s42003-024-05941-z (PMC10912366; doi:10.1038/s42003-024-05941-z)
Supplement: Supplementary file 2 — Description of Additional Supplementary Files [file 42003_2024_5941_MOESM2_ESM.pdf]

Description of Additional Supplementary Files

File name: Supplementary Data 1

Description: The source data behind the growth assay graphs in the paper
